# Vaccarin Regulates Diabetic Chronic Wound Healing through FOXP2/AGGF1 Pathways

**DOI:** 10.3390/ijms21061966

**Published:** 2020-03-13

**Authors:** Yixiao Liu, Jiangnan Sun, Xinyu Ma, Shuangshuang Li, Min Ai, Fei Xu, Liying Qiu

**Affiliations:** Department of Basic Medicine, Wuxi School of Medicine, Jiangnan University, Wuxi 214122, China

**Keywords:** vaccarin, diabetic chronic wounds, FOXP2, AGGF1, T1DM

## Abstract

Background: Diabetes mellitus is a growing global health issue nearly across the world. Diabetic patients who are prone to develop diabetes-related complications often exhibit progressive neuropathy (painless and sensory loss). It is usual for small wounds to progress to ulceration, which especially worsens with peripheral arterial disease and in the presence of anaerobic bacteria, culminating into gangrene. In our study, vaccarin (VAC), the main active monomer extracted from Chinese herb *vaccariae semen*, is proven to have a role in promoting diabetic chronic wound healing through a cytoprotective role under high glucose conditions. Materials and methods: We constructed a pressure ulcer on both VAC-treated and control mice based on a type 1 diabetes (T1DM) model. The wound healing index was evaluated by an experimental wound assessment tool (EWAT). We also determined the effect of VAC on the proliferation and cell migration of human microvascular endothelial cells (HMEC-1) by a cell counting kit (CCK-8), a scratch and transwell assay. Results: The results demonstrated that VAC could promote the proliferation and migration of high glucose-stimulated HMEC-1 cells, which depend on the activation of FOXP2/AGGF1. Activation of the angiogenic factor with G patch and FHA domains 1 (AGGF1) caused enhanced phosphorylation of serine/threonine kinase (Akt) and extracellular regulated protein kinases (Erk1/2). By silencing the expression of forkhead box p2 (FOXP2) protein by siRNA, both mRNA and protein expression of AGGF1 were downregulated, leading to a decreased proliferation and migration of HMEC-1 cells. In addition, a diabetic chronic wound model in vivo unveiled that VAC had a positive effect on chronic wound healing, which involved the activation of the above-mentioned pathways. Conclusions: In summary, our study found that VAC promoted chronic wound healing in T1DM mice by activating the FOXP2/AGGF1 pathway, indicating that VAC may be a promising candidate for the treatment of the chronic wounds of diabetic patients.

## 1. Introduction

Diabetic chronic wound is one of the most common complications of diabetic mellitus, which has become a growing worldwide health concern. It is hard to achieve effective results with conventional treatments, therefore it is of great significance to develop new treatment schemes [1]. Although the cause of diabetes chronic, non-healing wounds is multiple-cause, impaired endothelial function, poor local angiogenesis, and reduced blood supply are definitely critical factors [2]. Therefore, strategies aim to augment angiogenic responses in wound areas; for example, promoting vascular endothelial cell migration and proliferation may contribute to accelerate diabetic chronic wound healing.

*Vaccariae semen* is a kind of Chinese traditional medicine which has a function of stimulating blood circulation, eliminating edema, and promoting lactogenesis and diuresis [3]. Vaccarin (VAC) has been identified as the main monomer of *vaccariae semen* [4]. Current studies of VAC were mainly for the protective effects of endothelial cells. VAC could reduce hydrogen peroxide-induced apoptosis in human *EA·hy926* endothelial cells by inhibiting the *Notch* signaling pathway [5,6]. In vitro experiments revealed that VAC promoted proliferation of human microvascular endothelial cells (HMEC-1) by activating the fibroblast growth factor-2 (FGF-2) mediated fibroblast growth factor receptor (FGFR-1) signaling pathway under normal conditions [7]. Recently, it has been found that VAC could promote NO production via inhibition of the ROS/AMPK/miRNA-34a/eNOS signaling cascade [8], and protect HG-induced endothelial cell apoptosis by inhibiting the accumulation of reactive oxygen species and the expression of histone deacetylase 1 (HDAC1) [9]. In addition, bacterial cellulose–vaccarin (BC–VAC) membranes not only enhanced the mechanical and physical properties of the cellulose membrane but also promoted acute mechanical wound healing [10]. However, there is little information on the effect and mechanisms of VAC on chronic wound healing both in vitro and in vivo.

Forkhead box protein (FOXP) family is a transcription factor with a wing-like helical structure in the DNA binding region, and there are currently 17 subfamilies. As a member of the FOXP family, FOXP2 plays a role in promoting cell migration and invasion during tumor development [11,12]. FOXP2 could also promote AGGF1 expression at the transcriptional level, thus enhancing proliferation and migration of vascular endothelial cells exposed to glioma [13]. Recent research has indicated FOXP2 is essential in mediating the proliferation and function of islet α cells, suggesting that FOXP2 might play a role in diabetes mellitus [14]. Jia et al. reported that hyperglycemia downregulated FOXP2 and attenuation of endogenous FOXP2 resulted in a reduction of axonal growth [15].

Angiogenic factor with G patch and FHA domains 1 (AGGF1) is an angiogenic factor like VEGF. It was firstly described in Klippel–Trenaunay syndrome, which is highly expressed in vascular endothelial cells [16]. Chen and peers showed that AGGF1 increased angiogenesis and lumen diameter of veins, which was relevant to endothelial cell proliferation and migration [17,18,19]. Endothelial cells with overexpression of AGGF1 had significantly more capillary tube formation in a matrigel angiogenesis assay [18]. Therefore, the FOXP2/AGGF1 signaling pathway may be a potential intervention target under high glucose conditions.

The present study is thus designed to determine the effect of VAC on chronic diabetic wounds both in vivo and in vitro, in order to provide insights for potential therapeutic application of VAC for the healing of chronic diabetic wounds.

## 2. Results

### 2.1. Effects of Vaccarin on High Glucose-Induced Cell Viability

**Figure 1:**
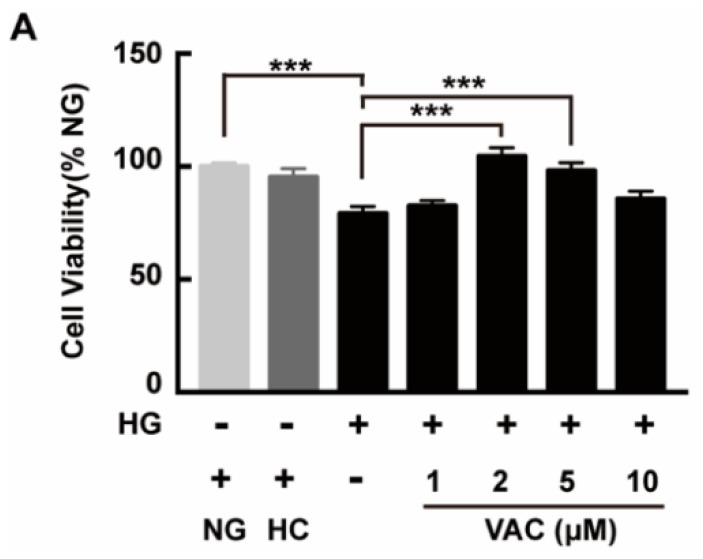
Vaccarin promoted proliferation on high glucose-exposed HMEC-1 cells. HMEC-1 cells were stimulated with 30 mM glucose (HG) for 24 h and then treated with various doses of VAC (0, 1, 2, 5, 10 μΜ) for 12 h. Cell viability was determined by CCK-8. Values are mean± SEM, *** *p* < 0.001, *n* = 6 for each group. NG, normal control group; HC, hyperosmotic control group; HG, high glucose group; VAC, vaccarin.

To investigate the effects of VAC on the viability of HMEC-1 cells after being stimulated with HG, a CCK-8 assay was used then. High glucose stimulation duration was determined based on peer works [20,21,22]. Results showed that the viability of HMEC-1 cells markedly decreased after being exposed to HG (*p* < 0.001), which was attenuated by VAC. Meanwhile, VAC treatment with a concentration of 2 μΜ conducted the maximum protective effect (*p* < 0.001, Figure 1). Simultaneously, there was no difference in cell viability between the NG (glucose 5 mM) group and HC (mannitol 30 mM) (*p* < 0.001, Figure 1).

### 2.2. Effects of Vaccarin on High Glucose-Induced Cell Migration

**Figure 2:**
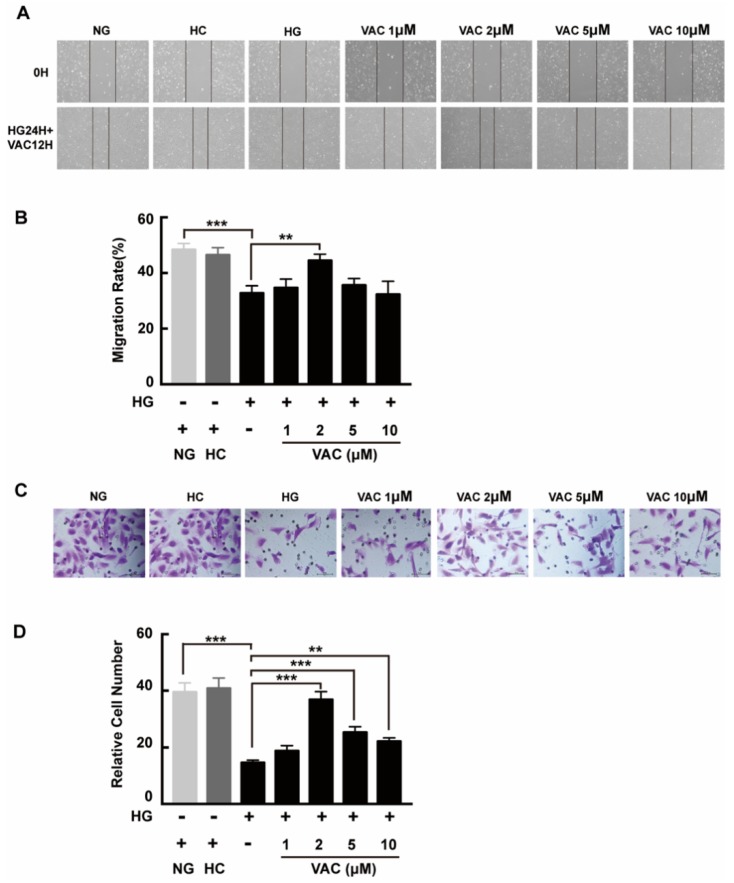
Vaccarin promoted migration on high glucose-exposed HMEC-1 cells. For scratch assay, cells were photographed by inverted microscope (40×) after scratched and VAC treatment. (**A**) The wound healing rate was measured by the closed area quantified by Image J software versus the initial area. (**B**) For transwell assay, the upper chambers were observed by inverted microscope (400×). (**C**) The migration ability was represented by the number of cells in the lower membrane. (**D**) Values are mean ±SEM, ** *p* < 0.01, *** *p* < 0.001, *n* = 5 for each group. NG, normal control group; HC, hyperosmotic control group; HG, high glucose group; VAC, vaccarin.

We next asked whether VAC could promote migration on high glucose conditions with a transwell assay and a scratch assay. Compared with the NG group, cell migration was obviously decreased in the HG group. Additionally, this phenotype was enhanced after VAC treatment. Similarly, there was no difference between the NC group and the NG group, while the wound healing rate of the HG group was apparently decreased (*p* < 0.01, Figure 2A,B). Statistical results demonstrated the VAC treatment with a dose of 2 μΜ had the most effective migration promoting effects (*p* < 0.01, Figure 2C,D).

### 2.3. Activation of FOXP2/AGGF1 Pathway in Response to Vaccarin Treatment

**Figure 3:**
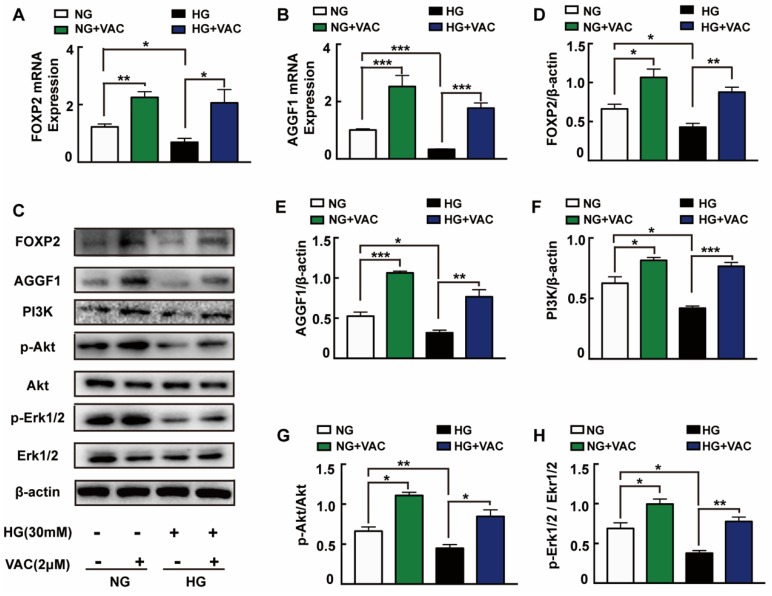
FOXP2/AGGF1 participated in the protective effects of vaccarin. HMEC-1 cells were stimulated with 30 mM glucose (HG) for 24 h and then treated with VAC (2 μΜ) for 12 h. Effects of VAC (2 μΜ) on the FOXP2 mRNA and AGGF2 mRNA expression in response to HG (**A**,**B**). Western blotting showing protein expressions of PI3K, p-Akt, Akt, p-Erk1/2, Erk1/2, FOXP2, and AGGF1 (**C**). Bar groups showing that the quantification of AGGF1/β -actin (**E**), FOXP2/β -actin (**D**), PI3K/β -actin (**F**), p-Akt/Akt (**G**), and p-Erk1/2/Erk1/2 (**H**). Values are mean ±SEM, * *p* < 0.05, ** *p* < 0.01, *** *p* < 0.001, *n* = 3 for each group. NG, normal control group; HG, high glucose group; VAC, vaccarin.

In order to explore the possible mechanisms responsible for VAC effects on cell migration, we detected the expression of PI3K, Akt, and Erk1/2 protein which have been reported closely associated with cell proliferation and migration. It was observed that PI3K expression, Akt and Erk1/2 phosphorylation were attenuated under high glucose (*p* < 0.05, Figure 3C,F–H). It was worth mentioning that VAC treatment obviously reversed HG-stimulated PI3K protein expression, Akt and Erk1/2 protein phosphorylation (*p* < 0.05, Figure 3C,F–H).

Through activating Akt and inhibiting reactive oxygen species generation, AGGF1 reduced the damaging effects of hyperglycemia on endothelial progenitor cells (EPCs) in vivo [23]. AGGF1 inhibited the NF-κB signaling via inactivation of the ERK pathway to lessen inflammation in a mouse hind limb ischemia model [24]. These studies indicate that AGGF1 may be an upstream regulatory protein of the PI3K/Akt and Erk1/2 pathways. Qianru He et al. reported that the silencing of FOXP1/FOXP2 downregulated the expression of AGGF1, reduced the viability, migration, and tube formation in U87 glioma-exposed endothelial cells [13]. We wondered whether VAC could promote PI3K/Akt and Erk1/2 phosphorylation and cell migration by regulating FOXP2/AGGF1. Subsequently, the mRNA and protein expression of FOXP2 and AGGF1 were detected. We found that HG strongly inhibited FOXP2 and AGGF1 expression both on transcription and protein levels (*p* < 0.05, Figure 3A–E), whereas being treated with VAC increased those expressions (*p* < 0.05, Figure 3C–E).

### 2.4. The Cell Migration Promoting Effects of VACCARIN Dependent on FOXP2

**Figure 4:**
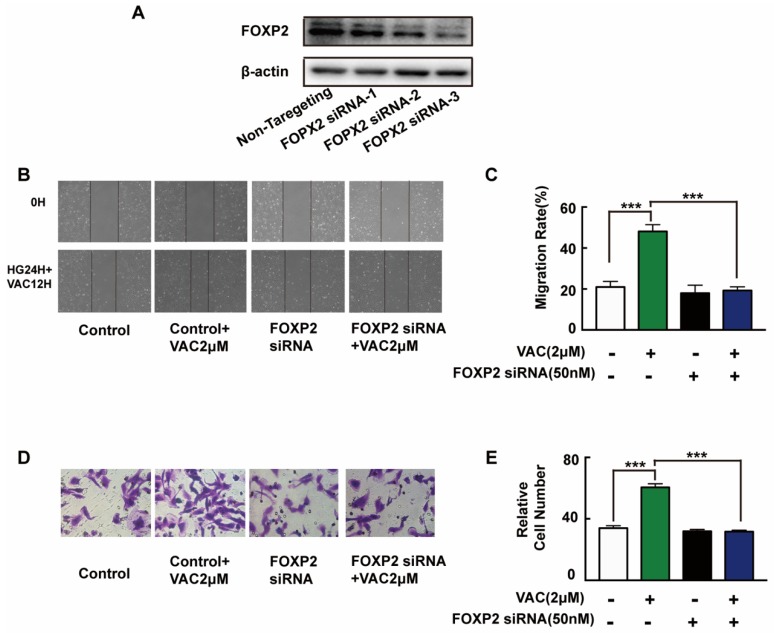
HMEC-1 cells migration after FOXP2 knockdown. HMEC-1 cells were incubated with FOXP2 siRNA for 6 h then replaced medium and cultured for another 48 h. Western blotting was used to test FOXP2 protein expression (**A**,**B**). HMEC-1 cells were pre-incubated with FOXP2 siRNA for 6 h then changed medium and cultured for another 12 h. Then cells were treated with HG (30 mM) for 24 h and VAC (2 μΜ) for 12 h. Scratch assay (**C**,**D**) and transwell assay (**E**) were performed to determine migration. Values are mean ±SEM, * *p* < 0.05, ** *p* < 0.01, *** *p* < 0.001, *n* = 3 for (**A**), *n* = 5 for (**B**–**E**). HG, high glucose group; VAC, vaccarin.

To further explore the mechanism of VAC promoted cell migration, siRNA was introduced to interfere with the expression of FOXP2 in HMEC-1 cells. The Western blotting results showed that the FOXP2 protein was silenced specifically and effectively by FOXP2 siRNA-3 (Figure 4A). Compared with the control group, cell migration of the VAC group was effectively enhanced (*p* < 0.001, Figure 4B–E), while cell migration was obviously decreased after FOXP2 knockdown (*p* < 0.001, Figure 4B–E). However, we noticed that FOXP2 siRNA had no effects on cell migration compared with the control group (*p* < 0.001, Figure 4B–E). The above results indicate that the effects of VAC on cell migration was obviously inhibited without FOXP2.

### 2.5. FOXP2/AGGF1 Mediated the Activation of PI3K/Akt and Erk1/2 Pathways Induced by Vaccarin

**Figure 5:**
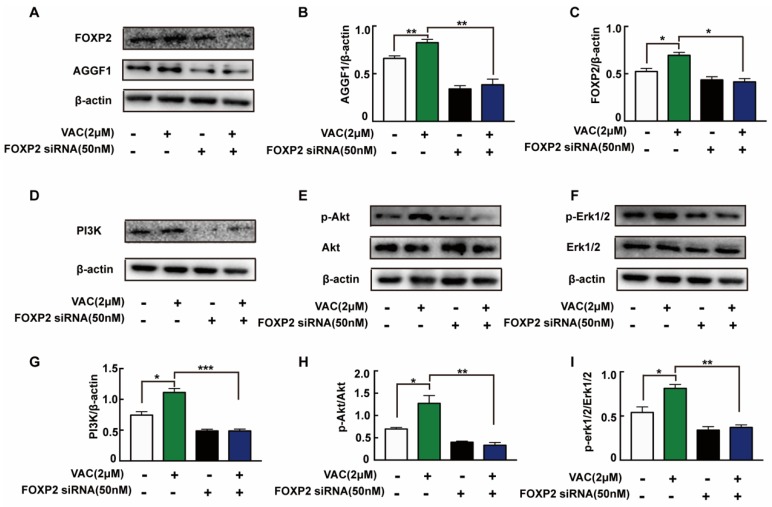
Expression of related proteins after knockdown of FOXP2. HMEC-1 cells were pre-incubated with FOXP2 siRNA for 6 h then changed medium and cultured for another 12 h. Then cells were treated with HG (30 mM) for 24 h and VAC (2 μΜ) for 12 h. Western blotting showing protein expressions of PI3K, p-Akt, Akt, p-Erk1/2, Erk1/2,FOXP2, and AGGF1 (**A**,**D**,**F**,**H**). Bar groups showing the quantification of AGGF1/β -actin (**B**), FOXP2/β -actin (**C**), PI3K/β -actin (**E**), p-Akt/Akt (**G**), and p-Erk1/2/Erk1/2 (**I**). Values are mean ±SEM, * *p* < 0.05, ** *p* < 0.01, *** *p* < 0.001, *n* = 3 for each group. VAC, vaccarin.

To confirm the relationship between FOXP2/AGGF1 and PI3K/Akt and Erk1/2, we detected relevant protein expression after siRNA interfered. Compared with the control group, the expression of AGGF1 protein in the VAC group was significantly increased, but there was no significant change in the FOXP2 knockdown group (Figure 5A–C). In VAC treated group, the expression of AGGF1 and phosphorylation of downstream proteins decreased significantly after FOXP2 knockdown (Figure 5D–I). The above results suggest that VAC regulated downstream migration-related protein expression by promoting FOXP2.

### 2.6. Effects of Vaccarin on Diabetic Wound Healing

**Figure 6:**
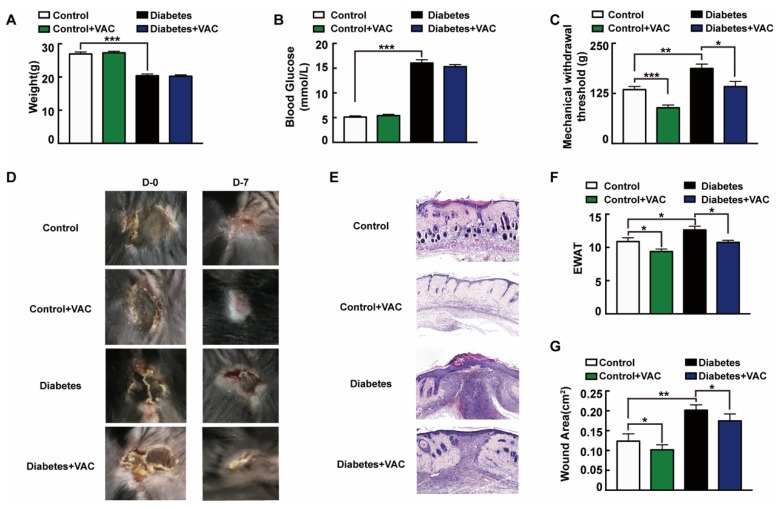
Vaccarin promotes diabetes chronic pressure ulcer healing. (**A**–**C**) The animal was sacrificed on day 7. Weight, tail mechanical threshold and fasting blood glucose were tested. (**D**) The wound was photographed on days 0 and 7 after treatment with VAC. (**E**) Representative photomicrographs of wound stained with H&E on day 7 after being sacrificed (100×). (**F**) EWAT assessments and (**G**) wound area on day 7. Values are mean ± SEM, *p* < 0.05, * *p* < 0.01, *** *p* < 0.001, *n* = 8 for each group. VAC, vaccarin; EWAT, experimental wound assessment tool.

We next asked whether the administration of VAC ameliorates chronic wound healing in T1DM mice. To confirm the effects of VAC on diabetic chronic wounds, we constructed an animal model of diabetic pressure ulcers. We found that the body weight slightly decreased and blood glucose strongly raised in the T1DM group (Figure 6A,B). Diabetic patients easily give rise to insensitive foot ulcerations because of peripheral nerve hypofunction, and those who inconveniently cannot move will also have insensitive pressure injuries [25]. The tail pressure Randall–Sellito test demonstrates a significant mechanical hypoalgesia in untreated diabetic mice, and the mechanical nociceptive threshold was enhanced in untreated diabetic mice, which could be restored in the VAC-treated groups (Figure 6C). The dorsal wound region was observed and photographed on days 0 and 7 after the ulcer formed. For the same stress stimuli, diabetic mice developed more severe skin ulcers than healthy mice. This suggested that the intrinsic physiological mechanisms of skin adaptive protection against ischemic stress had been impaired in diabetic mice [26]. As Figure 6D,E,G shows, VAC dramatically accelerates the wound healing rate. The wound almost closed completely on day 7 in the control group treated with VAC. EWAT assessment also confirmed the above results (Figure 6F).

### 2.7. Vaccarin Promoted Diabetic Chronic Wound Healing by Mediating FOXP2/AGGF1 Activation

**Figure 7:**
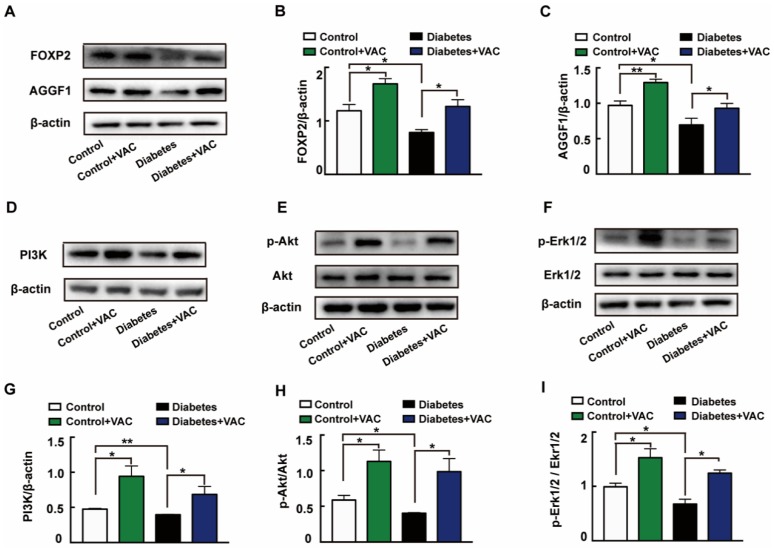
VAC promoted PI3K/Akt and Erk1/2 activation through the FOXP2/AGGF1 pathway to promote diabetic chronic wound healing. Animal wound skin tissues were extracted, lysed, and quantified. Western blotting was used to test relative protein expression. Western blotting showing protein expressions of PI3K, p-Akt, Akt, p-Erk1/2, Erk1/2, FOXP2, and AGGF1 (**A**,**D**,**E**,**F**). Bar groups showing that the quantification of AGGF1/β -actin (**B**), FOXP2/β -actin (**C**), PI3K/β -actin (**G**), p-Akt/Akt (**H**), and p-Erk1/2/Erk1/2 (**I**). Values are mean ±SEM, * *p* < 0.05, ** *p* < 0.01, *** *p* < 0.001, *n* = 3 for each group. VAC, vaccarin.

We next determined the molecular mechanism of VAC on diabetic chronic ulcers. In concordance with the in vitro experiments, the protein expressions of FOXP2 (Figure 7A,B), AGGF1 (Figure 7A,C), PI3K (Figure 7D,G), and phosphorylation levels of Akt (Figure 7E,H) and Erk1/2 (Figure 7F,I) were significantly decreased in diabetic wound skin tissues. These changes were all reversed by VAC. All these results demonstrate that VAC accelerates diabetic chronic wound healing through the FOXP2/AGGF1 pathway to promote PI3K/Akt and Erk1/2 activation.

## 3. Discussion

Endothelial cell migration is one of the important signs in angiogenesis, and also the early steps of angiogenesis cascade reaction [27]. Therefore, protecting the endothelial function to promote vascular reconstruction in wound healing has become a promising therapeutic target [28]. In the process of angiogenesis, capillary buds are formed by the proliferation and migration of vascular endothelial cells to surrounding tissues. Endothelial cells can reshape and expand the vascular network in almost all tissues of the human body by migration [29]. Diabetes can significantly interfere with endothelial function, disrupt the normal wound healing, tissue regeneration, and recovery of healthy vascular systems so that diabetic wounds affected will show reduced numbers of blood vessels and capillary density [30]. Numerous studies have emphasized the lack of angiogenesis and blood supply in diabetic wound healing [31]. Further, poor angiogenesis and lack of a well-perfused vascular bed will caused a delay in wound healing. Therefore, it is extremely important to promote endothelial function and vascular reconstruction during the healing of diabetic chronic wounds. In our study, we found that VAC accelerated the wound healing of T1DM mice in vivo and promoted microvascular endothelial cell proliferation and migration under high glucose conditions in vitro. The above results demonstrate the potential benefits of VAC on endothelial function and microvascular growth.

PI3K/Akt and Erk1/2 pathways have been proven to play an important role in cell migration. Downstream of the phosphatidylinositol 3-kinase (PI3K) signaling pathway, serine/threonine kinase (Akt) is primarily involved in cell survival, cell proliferation, and plays a role in mediating cell migration. Zhang et al. have reported that notoginsenoside Ft1 from *Panax notoginseng* promoted fibroblast proliferation via the PI3K/Akt/mTOR signaling pathway and benefited diabetic chronic wound healing [32]. Ku Y.H. et al. reported that rosiglitazone significantly promoted endothelial cell migration via the activation of PI3K/Akt [33]. Extracellular signal-regulated kinase (Erk1/2), part of the MAPK family, is located downstream of the Mek signaling pathway. In endothelial cells, the Erk signaling pathway played a significant role in proliferation, differentiation, migration, senescence, and apoptosis [34]. Reports have shown hepatocyte growth factor (HGF) treatment induced activation of the MAPK/Erk pathways in human myoblasts, further promoting cell migration [35]. Meanwhile, inhibition of Akt and Erk significantly blocked fibroblast proliferation and migration [36]. Our previous study had demonstrated that VAC significantly boosted endothelial cell proliferation and migration in vitro in normal glucose conditions [7]. In the present study, our results demonstrate that VAC treatment could reverse the proliferation and migration of HMEC-1 cells inhibited by HG, and stimulate the phosphorylation of PI3K/Akt and Erk1/2 in HMEC-1 cells and diabetic mice wound skin tissues. These results indicate that VAC might activate PI3K/Akt and Erk1/2 pathways to promote cell proliferation and migration.

Studies have found that AGGF1 could activate Akt and inhibit the production of reactive oxygen species, thus reversing all the destructive effects of hyperglycemia on endothelial progenitor cells [23]. AGGF1 could promote angiogenesis in glioma-exposed endothelial cells, which was regulated by FOXP2 at the transcription level [13]. FOXP2 could promote tumor invasion and migration. The knockdown of FOXP2 attenuated the proliferation and invasion of triple-negative breast cancer, inhibited tumor progression, and metastasis [37]. Those results indicate that FOXP2 also plays an important role in cell migration. However, the expression of FOXP2 and AGGF1 in diabetic chronic wounds have not yet been elucidated. In our experiment, we found that VAC demonstrated the ability to activate the FOXP2/AGGF1 signaling pathway, thereby stimulating the phosphorylation of PI3K/Akt and Erk1/2 in HMEC-1 cells under high glucose. It is worth mentioning that a knockdown of FOXP2 inhibited the proliferation and migration effect and phosphorylation of PI3K/Akt and Erk1/2 in HMEC-1 cells. Therefore, it could explain that in the process of HG-inhibited angiogenesis and wound healing, FOXP2/AGGF1 is a potential pathway in which VAC promotes endothelial function.

Based on these studies, we prove that vaccarin (VAC) further promotes endothelial cell migration and wound healing by promoting the expression of the FOXP2/AGGF1 pathway to activate downstream PI3K/Akt and Erk1/2 signaling pathways. Peer works found that VAC had a better therapeutic effect on blood glucose with a dose of 1 mg/kg (i.p.) for 4 weeks [8,38]. However, we had discovered that VAC could promote diabetic chronic wound healing with a lower dose (0.4 mg/kg, i.p.) and in a much shorter period (7 days) under a hyperglycemia condition but without reduction of blood glucose. Taken together, our results show for the first time that VAC antagonizes HG-induced stimulation on HMEC-1 cell through regulating the FOXP2/AGGF1 pathway. These results indicate that FOXP2 is one of the targets of VAC, and it also proves that FOXP2 plays an active role in chronic wounds of diabetes, enriched the clinical evaluation system for chronic wounds.

## 4. Materials and Methods

### 4.1. Reagents and Chemicals

Vaccarin was purchased from Shanghai Shifeng Technology (Shanghai, China). Streptozocin (STZ) was acquired from Sigma (St. Louis, MO, USA). FOXP2, AGGF1, total Akt, phospho-Akt, total Erk1/2, phospho-Erk1/2 antibodies were bought from Abclonal (Wuhan, Hubei, China). Total PI3K, β-actin antibodies were purchased from Abcam (Cambridge, MA, USA). The specific primers, control siRNA, and targeted FOXP2 siRNA were synthesized by Shanghai Sangon Biotech Co. Ltd. (Shanghai, China).

### 4.2. Cell Culture and Treatments

Human microvascular endothelial cells (HMEC-1) were provided by the U533 Institute of the French National Institute of Health Medicine and were cultured in MCDB131 (Sigma St. Louis, MO, USA) containing 5 mM glucose, 10% FBS (Lonsera, Shuangru Biotech, Shanghai, China), 10 ng/mL EGF (Proteintech, Inc, Wuhan, China), and 100 U/L penicillin and 100 μg/mL streptomycin (Gibco, Carlsbad, CA, USA). The cells were incubated at 37 °C under an environment with 5% CO_2_. When grown to a logarithmic growth phase, HMEC-1 cells were seeded into proper plates and exposed to HG (30 mM glucose) or mannitol (30 mM) for 24 h and then treated with vaccarin (2 μM) for 12 h.

### 4.3. Cell Proliferation Assay

The cell proliferation of HMEC-1 cells was examined by the Cell Counting Assay Kit-8 (CCK-8). HMEC-1 cells seeded into 96-well plates were exposed to HG or mannitol for 24 h and then treated with vaccarin (1, 2, 5, 10 μM) for 12 h. Cells were incubated with CCK-8 for 1 h and a Biotek microplate reader (Winooski, VT, USA) was used to measure absorbance at 450 nm.

### 4.4. In vitro Wound Healing Assay

The wound healing rate of HMEC-1 cells was tested by an in vitro wound healing assay. HMEC-1 cells were seeded into 6-well plates. When the cells grew up to 90% confluence, a linear wound gap was scraped using a new 200 uL pipette tip. Cell debris was washed out by phosphate buffer saline (PBS) and new medium with different doses of vaccarin were added into wells. Wound healing condition was acquired by an inverted microscope (Nikon, Tokyo, Japan) at a magnification of 40× The wound healing rate was quantified by the wound area with Image J software 1.0 (National Institutes of Health, MD, USA).

### 4.5. Transwell Assay

Transwell assay was carried out to observe cell migration at a 3D level. After being exposed to HG or mannitol for 24 h, the HMEC-1 cells were trypsinized and seeded into the upper chamber (8.0 μM pore size, Corning Costar Corporation) with vaccarin (1, 2, 5, 10 μM) and serum-free MCDB131 medium. The lower chamber was added with 750−μL MCDB131 medium. Then, 12 h later, cells in the upper chamber were removed by a cotton swab. The chambers were washed twice with PBS and fixed with 4% paraformaldehyde for 30 min. After washing twice with PBS again, the chambers were stained with 5% crystal violet stain for 30 min. Cell migration was observed by an inverted microscope (Nikon, Tokyo, Japan) at a magnification of 400×. Image J software 1.0 was used to calculate the number of migrated cells.

### 4.6. Real-Time PCR Analysis

The FOXP2 and AGGF1 mRNA expression were detected by quantitative polymerase chain reaction. Total RNA was extracted by Trizol Reagent (Cwbio, Nanjing, China). RNA (1 μg) was used to generate cDNA with ReverAid First Strand cDNA Synthesis (Thermo Fisher Scientific Inc., Massachusett, USA). After the real-time quantitative PCR was performed with cDNAs and gene-specific primer pairs with Hieff UNICON Power qPCR SYBR Green Master Mix (Yeasen, Beijing, China) by a fluorescence quantitative LightCycler 480 Real-Time PCR system (Roche, Basel, Swenden). 2^−ΔΔCT^ was used to show the fold change. The primers for FOXP2: 5′- AAG CAT GCT GGC TCA GTC TT -3′ (Forward), 5′- CAC AGG CAC TGC AAA TGT GTT -3′ (Reverse). The primers for AGGF1: 5′- AGC TGG AAA ACG TAG GGA GC -3′ (Forward), 5′- GAA GCT GGA TCG GCG TTT TC -3′ (Reverse). The primers for β-actin: 5′- CAC CAT TGG CAA TGA GCG GTT CC -3′ (Forward), 5′- GTA GTT TCG TGG ATG CCA CAG G -3′ (Reverse).

### 4.7. Western Blotting

The total protein was extracted by RIPA lysis buffer (Cwbio, Nanjing, China) with protease and phosphatase inhibitor (Cwbio, Nanjing, China) as peer-described [9] and quantified with BCA protein assay kit (Beyotime Biotechnology, Shanghai, China). Equal amounts of protein were electrophoresed, blotted, and incubated with the required antibodies at 4 °C overnight. Then, membranes were washed by TBST and incubated with the appropriate horseradish peroxidase-conjugated antibodies. The blots were visualized by a chemiluminescence detection system (Millipore Demonstrate, MA, USA). Signal intensity quantified by Image J was used to show the activated protein expression.

### 4.8. siRNA Interference

Cells were resuspended in fresh medium without antibiotics and transfected separately with small interference RNA (siRNA) sequences against FOXP2 (50 nM) or a control siRNA (50 nM) using Lipofectamine™ 2000 (Invitrogen, Carlsbad, CA, USA) according to the manufacture’s protocols. The siRNA sequences targeted FOXP2 were as follows: sense, 5′-GCA GCA GAU CCU UCA GCA ATT-3′; antisense, 5′-UUG CUG AAG GAU CUG CUG CTT-3′. The control siRNA sequences were as follows: sense, 5′-GUA UGA CAA CAG CCU CAA GTT-3′; antisense, 5′-CUU GAG GCU GUU GUC AUA CTT-3′

### 4.9. Animal Model and Treatments

Male C57BL/6J mice aged 6 to 8 weeks were purchased from the Model Animal Research Center of Nanjing University and housed in a 12 h light–dark cycle with free access to food and water. All of the experiments were confirmed by the Experimental Animal Care and Use Committee of Jiangnan University in 15th October 2018 (JN. No.20181015c0401122[215]). The experimental procedures were carried out according to the Guide for the Care and Use of Laboratory Animals published by the US National Institute of Health (NIH publication, 8th edition, 2011). Mice were randomly divided into 4 groups (*n* = 8). In two groups, diabetic mice were induced by injection of streptozotocin (STZ) (160 mg.kg^−1^, i.p.), while the other two groups received an injection of vehicle (citrate buffer, pH = 4.5). After 6 weeks, ulcer formation was inducted by magnet pressure. Three days after, one from the control group and one from the diabetic group received a vaccarin (0.4 mg.kg^−1^, i.p.) injection. The other two groups were treated with vehicle (0.9% NaCl, i.p.).

### 4.10. Pressure Ulcer Model

The dorsal hair was shaved by a pet shaver the day before. After anesthetizing the mice with 1% sodium pentobarbital, the back skin of the mice (including the epidermis, dermis, and subcutaneous tissue) was clamped in two cylindrical magnets (10 mm diameter and 3 mm thick, with an average weight of 0.5 g and 1600 Gauss magnetic force) (Oulex, Ningbo, China) for 8 h. This process was repeated twice to achieve the effect of simulating ischemia-reperfusion injury. Reports have shown that exposure to pressure during 12 hours could reproducibly elicit a skin ulcer in normal mice [39].

### 4.11. Pressure Ulcer formation Assessments

The wound area was photographed on days 0 and 7. Wound healing condition was scored by the experimental wound assessment tool (EWAT) [40]. On day 7, the mice were sacrificed after anesthesia with 1% pentobarbital and skin tissue samples were dissected from the mice. The tissues were fixed in 4% paraformaldehyde, embedded in paraffin, and sectioned. Sections were stained with hematoxylin and eosin and observed under Pannoramic SCAN (3DHISTECH Ltd., Budapest, Hungary).

### 4.12. Mechanical Pressure Algesia

Tail pressure thresholds were registered with a tail tenderness meter (Ruanlong, Shandong, China) for the Randall–Selitto test (increase the pressure at a constant rate and record the number of grams of pressure in the event of a squat or bite). We performed three tests at least 15 min apart for each animal and calculated the average values of these tests.

### 4.13. Statistical Analysis

All results were expressed as mean ± SEM from at least three independent experiments. *T*-test was used to accomplish the analysis of statistics for the comparisons between two groups. For multiple group comparisons, statistical analysis was performed by ANOVA followed by Dunnett’s test. *p*-value <0.05 was taken as significant.
